# POSITIVE CAREGIVING AND CAREGIVING RELATIONSHIP ASSOCIATION WITH MENTAL HEALTH AND PERCEIVED GENERAL HEALTH

**DOI:** 10.1093/geroni/igac059.2057

**Published:** 2022-12-20

**Authors:** Cristina de Rosa, Ashleigh Holmes, Weijun Wang, Yu-Ping Chang

**Affiliations:** University at Buffalo, Buffalo, New York, United States; University at Buffalo, Buffalo, New York, United States; University at Buffalo, Buffalo, New York, United States; University at Buffalo, Buffalo, New York, United States

## Abstract

Caregiver burden is well understood as an important contributor to caregiver health. However, little is known about how positive aspects of caregiving (i.e., personal growth, gratitude, finding meaning) and the quality of caregivers’ relationships with care recipients might play a role in caregiver health. The study aimed to examine whether positive caregiving and caregivers’ relationship with care recipients were associated with caregiver mental health (depression and anxiety) and perceived general health. The sample consisted of 2,652 family caregivers in the National Study of Caregiving (NSOC) III (2017) providing care to older adults. A series of multiple regression models with covariate adjustments (i.e., caregiver’s age, sex, and race/ethnicity) were performed to examine the associations. Results indicated that positive aspects of caregiving predicted caregiver mental health but did not predict perceived general health. Caregivers’ relationship with care recipients and caregiver burden significantly predicted caregiver mental health (b = 0.285 [S.E. = 0.045], p < .001) and perceived general health (b = 0.096 [0.016], p < .001). After controlling for caregiver burden, only caregivers’ relationship with care recipients remained a significant predictor of caregiver mental health (b = 0.182 [0.041], p < .001) and perceived general health (b = 0.077 [0.018], p < .001). Our results suggest that positive caregiving perceptions and quality of relationships between caregivers and care recipients are linked to better caregiver mental health. Interventions to reduce caregiver burden, including strategies to help caregivers maintain positive attitudes and positive relationships with care recipients, might be beneficial to improving caregiver health.

